# Comparing Temporal Averaging and Transformer Encoders for Video-Based Cattle Behavior Classification Under Severe Class Imbalance

**DOI:** 10.3390/vetsci13101068

**Published:** 2026-10-10

**Authors:** Daniyar Pirlepesov Maratovich, Dae-Yeong Kang, Mohammad Ismail, Usama Hanif, Seong-hun Kim, Dasanayaka Lekamalage Vindya Sathsarani Dasanayaka, Reginald Gbenga Azuatalam, Muhammad Ismail Khan, Hyeon-Tae Kim

**Affiliations:** 1 Department of Smart Farm, Gyeongsang National University, Jinju 52828, Republic of Korea; daniyar@gnu.ac.kr (D.P.M.); dykang@gnu.ac.kr (D.-Y.K.); tjdgnszhd@naver.com (S.-h.K.); vindy@gnu.ac.kr (D.L.V.S.D.); 2 Department of Bio-Systems Engineering, Gyeongsang National University, Jinju 52828, Republic of Korea; ismail@gnu.ac.kr (M.I.); usama@gnu.ac.kr (U.H.); reginald@gnu.ac.kr (R.G.A.); 3 Department of Computer Science, Bahria University, Shangrila Road, Sector E-8, Islamabad 44000, Pakistan; affablezaki@gmail.com; 4 Department of Bio-Industrial Machinery Engineering, Institute of Smart Space Agriculture (ISSA), Gyeongsang National University, Jinju 52828, Republic of Korea

**Keywords:** edge deployment, probabilistic calibration, rare-class analysis, stratified cross-validation, precision livestock farming

## Abstract

Automated cattle behavior monitoring from video is challenging due to severe class imbalance and the need for efficient temporal modeling. We compared four architectures on 542 videos containing standing, lying down, and rumination; rumination appeared only six times, while two dataset classes were absent. ResNet-50 and EfficientNet-B3 backbones were paired with either a Transformer encoder or temporal averaging and trained with three-fold stratified cross-validation and weighted cross-entropy. EfficientNet-B3 with temporal averaging (Eff-B3 + Avg) achieved the highest out-of-fold accuracy (85.61%) and best calibration (ECE = 0.074), while requiring 41 MB and 28 ms per video. Its Transformer counterpart scored 84.13% despite four times more parameters. ResNet-50 + Transformer reached 84.69% but was poorly calibrated and storage-intensive. Frame ablation identified 16 frames per video as the best accuracy-speed trade-off. No model correctly classified any rumination example, underscoring that rumination was not learnable under the specific weighted-cross-entropy and stratified-sampling strategies evaluated here; this finding does not establish a general impossibility of learning extremely underrepresented behaviors under alternative training strategies such as focal loss, oversampling, anomaly detection, or self-supervised pre-training. Rare-class analysis, treating support ≤106 as rare, showed high-confidence rumination errors: mean confidence was 0.891, but only 1.01% of probability mass was assigned to the true class, yielding a class-wise expected calibration error of 0.891. These findings indicate that calibration post-processing cannot rescue a class with zero true positives.

## Simple Summary

Monitoring cattle behavior from video could reduce the need for continuous manual observation. However, models that recognize common behaviors may still fail on behaviors with few training examples. This study compared four computer models using 542 videos labeled mainly as standing or lying down, with only six labeled as rumination, or chewing cud. The models combined information from video frames either through averaging or through a more complex method that learns relationships between frames. The best-performing model used averaging and correctly classified approximately 86% of the evaluated videos, while requiring less computer storage than the more complex alternatives. None of the models correctly recognized rumination, and the best-performing model made these errors with high confidence. These findings show that overall accuracy alone can conceal failure on underrepresented behaviors. More training examples of these behaviors and testing across different farms are needed before such models can reliably support comprehensive cattle behavior monitoring.

## 1. Introduction

Monitoring cattle behavior is a part of taking care of livestock. Just by watching what a cow does like how it stands or lies down we can tell a lot about its health and wellbeing. For example, if a cow is lying down often it might be getting sick [1,2]. For example, if it is not eating much, it might have stomach problems [3]. Farmers used to watch their cows but this is a lot of work and hard to do with a lot of cows [4].

Technological advancements like accelerometer ear tags that track movement have made it easier to monitor cows. However, these tags cannot explain the difference between behaviors like standing still or lying. These ear tags also fail to provide information about the animal’s surroundings. Nowadays, low-cost cameras and recent advancements in computer vision have made animal monitoring relatively easy. Such technological solutions cannot just capture animal posture but can carefully observe the environmental interaction as well [5].

However, animal monitoring using cameras on a farm comes with several challenges. For example, the light conditions on the farms are not always the same; the camera angles may not fully cover the barn area; the critical class imbalance issue, since most of the coverage involves standing and lying down. The dataset assembled for this study makes the imbalance problem concrete rather than abstract. Standing accounts for 326 of the 542 collected videos; lying down for 199. After that the numbers drop sharply, i.e., rumination appears in 13 clips, foraging in 4, and drinking water not at all. The class distribution observed in the present corpus depends on the recording strategy (single fixed camera, limited recording duration), camera configuration (one elevated vantage point covering the resting area), annotation procedure (per-frame labeling followed by dominant-label consolidation), and the dominant-label consolidation rule itself. We therefore cannot definitively attribute the observed distribution to natural behavioral prevalence rather than to the sampling and preprocessing pipeline. The present distribution should accordingly be interpreted as a realistic lower-bound benchmark for what a fixed single-camera deployment would capture under similar recording conditions, rather than as a representative sample of natural cattle behavioral prevalence. Generalization to other farms, camera configurations, or annotation protocols cannot be inferred from this single-farm dataset and is identified in Section 4.4 (Limitations) as a boundary on the present claims.

Recognition of cattle behavior from camera footage did not begin with deep learning, and understanding why earlier approaches stalled is useful for framing what the present architectures are actually solving. Throughout the late 2000s and into the following decade, the standard engineering approach was to decompose the problem into two sequential stages: first, extract a numerical descriptor that captured some property of the image believed to be diagnostic, e.g., motion magnitude through optical flow, local edge orientation through HOG vectors, pixel co-occurrence statistics through texture matrices; and second, train a classical classifier on those numbers [5]. The two-stage design was not naïve. It reflected genuine knowledge about what visual properties correlate with behavior in structured settings. The difficulty was that the correlation was fragile. Handcrafted features often failed to generalize under real-world conditions. Variations in coat color or camera position reduced the reliability of texture and shape-based descriptors, limiting their ability to capture behavior consistently. Varying barn lighting across the day introduced additional instability, gradually weakening the correspondence between measured feature values and the correct behavior labels. The underlying problem shared across all these failure modes was the same: each descriptor encoded a fixed assumption about which image properties were diagnostic, and once farm conditions broke that assumption, no downstream classifier, regardless of its sophistication, could recover the information that had been lost. The classifier could only work with whatever the descriptor produced, and if the descriptor had stopped measuring what it was designed to measure, classification accuracy declined no matter how capable the model was.

Unlike handcrafted pipelines where a researcher had to decide in advance which image statistics to compute, convolutional neural networks approached feature extraction differently; for example, the network itself learned which spatial patterns were predictive by exposure to large numbers of labeled examples. Gradient-driven weight updates during training determined what to extract from images, rather than explicit human design. The features that emerged from this process were not pre-specified; they were discovered through optimization. AlexNet’s 2012 ImageNet success established the superiority of learned feature representations over handcrafted features [6]. Subsequent architectures improved performance through deeper networks (VGG), efficient convolutions (Inception), and residual connections (ResNet), while enabling transfer learning across diverse tasks [7].

Frame-wise classification is effective for static behaviors such as standing and lying but fails to capture subtle temporal patterns like rumination. To address this, spatiotemporal models such as C3D and I3D jointly learn spatial and temporal features, enabling robust motion-based behavior recognition [8,9]. However, their high computational and memory requirements limit their practicality for livestock monitoring on resource-constrained edge devices [10].

The Transformer’s entry into computer vision came by way of natural language processing, where the mechanism of learned pairwise attention had already demonstrated that any two elements in an input sequence could be directly related without imposing a fixed computational path between them, a flexibility that proved transformative for machine translation and language understanding [11]. This flexibility is valuable for behavior whose diagnostic cues are spread unevenly across a clip. Transformer-based video models, including hybrid CNN + Transformer designs in which a convolutional backbone extracts per-frame spatial features and a Transformer aggregates them temporally, have become a leading paradigm for human action recognition [12] and are now finding application in livestock settings. A TimeSformer baseline applied to beef cattle across five behavior categories yielded 90.33% mean accuracy [13]. The ARTEMIS multimodal framework extended Transformer-based recognition to a large animal-kingdom benchmark [14], and a YOLO + TimeSformer pipeline for dairy digital-twin applications achieved 85.0% overall accuracy [10]. What none of these studies examine is a dataset in which one class is structurally absent from training, i.e., a scenario that is commonplace on real farms where certain behaviors are rarely captured by chance.

Class imbalance touches every practical deployment challenge. Resampling either duplicates minority examples (over-sampling) or discards majority ones (under-sampling), risking overfitting or information loss [15]. Synthetic methods like SMOTE work for tabular data but are hard to apply to video [15]. Cost-sensitive learning penalizes minority-class errors without changing the data distribution [16]. Focal loss reshapes the cross-entropy surface so that well-classified examples contribute less to the gradient [17]. Related loss functions for imbalanced classification, including variants of focal loss, are reviewed in a general survey of loss functions and metrics in deep learning [18]. Self-supervised pre-training on unlabeled video has emerged as a complementary strategy that learns generalizable representations without requiring large labeled sets [19]. Among these options, weighted cross-entropy combined with stratified cross-validation has the appealing property that it is data-preserving, easy to implement, and directly tied to the empirical class frequencies within each training fold, making it well matched to the constraint that all available data must be used when samples are scarce. Ref. [13] adopted explicit data augmentation and oversampling in their beef-cattle study; Ref. [20] approached rare aggressive pig behavior as an anomaly-detection problem. However, a rigorous evaluation of weighted cross-entropy specifically in the setting where one class is entirely missing has not been reported for cattle behavior recognition.

Validation design is a related but distinct concern. A single fixed train-test split is vulnerable to the particular way data happen to be divided: if one fold happens to contain the few rumination clips, a model trained without them and tested on them will look far worse than a model that had the opposite luck. Stratified k-fold cross-validation preserves class distributions across folds, providing more reliable performance estimates while reducing split bias and the risk of data leakage from correlated livestock videos [21,22]. When repeated measurements from the same animal or recording session are present, grouped (per-animal or per-session) cross-validation provides a stronger test of biological independence than video-level splitting [23].

Probabilistic calibration remains underexplored in livestock video analysis. Well-calibrated confidence scores improve decision-making by indicating when automated predictions can be trusted and when manual verification is required. Empirical work on calibration measurement in deep learning has shown that the choice of calibration measure can change how models are ranked and recommends conditioning on the class rather than relying on global expected calibration error (ECE) alone [24]. A recent survey cataloging deep learning methods for animal behavior analysis [25] does not address whether per-frame predictions are temporally consistent or whether confidence scores are meaningfully calibrated. Metrics relevant to these questions, including per-frame prediction entropy, rolling standard deviation of confidence across frames, and reliability diagrams plotting mean confidence against observed accuracy, are computationally cheap and interpretable without specialist knowledge. Their near-complete absence from livestock video evaluation studies represents a practical gap, since confidence miscalibration directly affects how monitoring outputs should be acted upon, and this study takes a step toward addressing it.

The preceding state-of-the-art review identifies four connected knowledge gaps. First, previous livestock video studies rarely compare Transformer-based temporal encoding directly with simple temporal averaging while holding the spatial backbone fixed. Second, the interaction between backbone quality and temporal aggregation remains unclear under severe class imbalance. Third, existing evaluations seldom examine whether cross-validation stability translates into stronger pooled out-of-fold performance. Finally, temporal consistency, probabilistic calibration, and failure on extremely rare or absent behavior classes remain insufficiently studied in realistic farm datasets.

The central hypothesis of this study is that, for posture-dominated cattle behaviors, a strong convolutional backbone combined with temporal averaging can match or outperform a more complex Transformer encoder while requiring fewer parameters, less storage, and lower inference time. We further hypothesize that weighted cross-entropy and stratified cross-validation can reduce majority-class bias but cannot compensate for a behavior class represented by only a handful of examples.

To test these hypotheses, this study pursues a single consolidated objective: to identify, through cross-validated comparison under severe class imbalance, the architecture that achieves the most favorable trade-off among classification accuracy, computational efficiency, probabilistic calibration, and rare-class behavior on the present CBVD-5 video corpus. This single objective encompasses the comparison of temporal-averaging and Transformer-based aggregation under matched backbones, the assessment of EfficientNet-B3 versus ResNet-50 spatial representations, the joint evaluation of fold-wise stability and pooled out-of-fold performance, and the determination of deployment suitability for resource-constrained edge devices. Consolidating the previously enumerated sub-objectives into a single statement reflects the structure of the reported analysis, in which these four aspects are evaluated jointly rather than sequentially.

This research advances the state of the art through a controlled comparison of backbone and temporal-aggregation choices under naturally severe class imbalance. In addition to accuracy and efficiency, it evaluates calibration, temporal stability, frame-count sensitivity, and rare-class failure, thereby providing a deployment-oriented assessment that is largely absent from previous livestock behavior-recognition studies.

## 2. Materials and Methods

**Figure 1:**
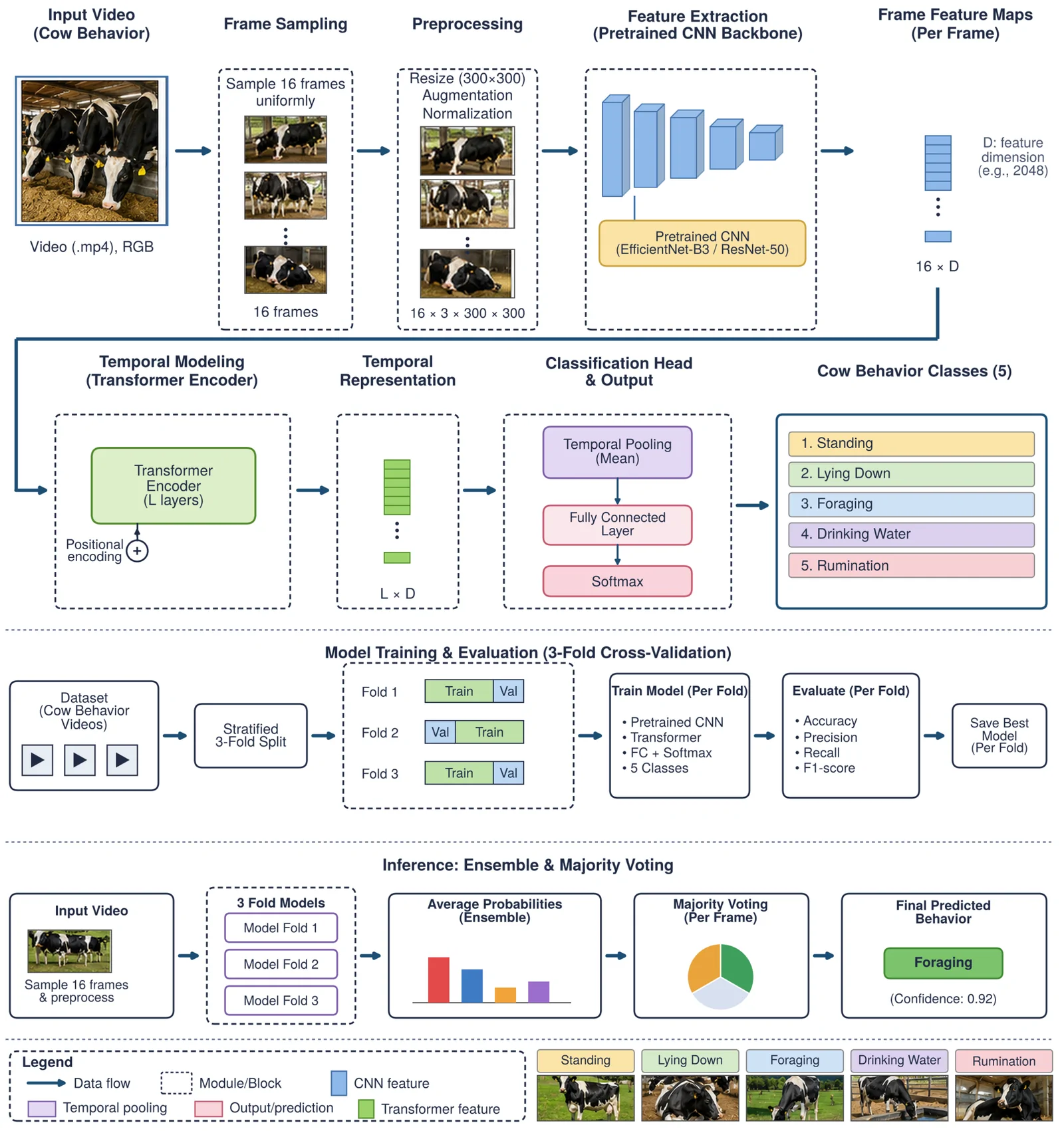
Workflow diagram of the proposed video-based cattle behavior classification pipeline. Colors identify block types as defined in the legend at the bottom left: data flow, module/block, convolutional neural network (CNN) feature, Transformer feature, temporal pooling, and output/prediction. Vertical dots indicate repeated frames or feature vectors that are omitted for brevity; no content is missing.

A schematic workflow of the complete pipeline is presented in Figure 1, starting from raw video input through frame sampling, feature extraction, temporal modeling, and cross-validated training to ensemble inference.

### 2.1. Dataset and Preprocessing

This subsection describes how the raw CBVD-5 annotations were consolidated into video-level behavior labels and prepared for model training. It explains the class distribution, the stratified cross-validation strategy, and the treatment of severe imbalance across the observed behaviors. It also details the uniform frame-sampling and image-normalization procedures used to produce consistent model inputs.

#### 2.1.1. Dataset Description

This subsection describes the CBVD-5 video dataset, the recording configuration, the criteria by which the original 687-video collection was reduced to the 542 analyzed videos, the dominant-label procedure by which the original five behavior categories were consolidated into three classes with non-zero support, the stratified cross-validation strategy, and the treatment of severe class imbalance across the observed behaviors. It also details the uniform frame-sampling and image-normalization procedures used to produce consistent model inputs.

This study utilized the CBVD-5 video dataset. Table 1 traces how behavior labels evolved through five successive processing stages, from the original frame-level annotations through to the final evaluation metrics reported in the classification output. At the raw annotation stage, the dataset contained 34,102 training rows and 3761 validation rows spanning four behavior classes. Once each video was assigned its dominant label based on whichever class appeared most frequently in its frame-level annotations, the dataset consolidated to 542 unique videos having 330 labeled as standing, 206 as lying down, 6 as rumination, and zero for both foraging and drinking water. These 542 videos were then divided into three stratified folds for cross-validation, and out-of-fold predictions were generated for every video exactly once; that is, each clip was scored by the best checkpoint from the fold in which it served as held-out data. The resulting 542 OOF predictions carry support values of 330, 206, and 6 for standing, lying down, and rumination respectively, numbers that match the video-level counts exactly and confirm that the pipeline from raw annotations through to final evaluation metrics was fully consistent and traceable at every stage.

Recording configuration, animal coverage, and cross-validation independence. The original CBVD-5 recordings were captured by a single fixed-position ceiling-mounted camera installed at an elevated vantage point in a dairy barn, overlooking the primary resting and standing areas. According to the publicly available dataset documentation on Kaggle, a single camera angle was used throughout the recording period to ensure viewpoint consistency, and the same herd of dairy cattle was filmed continuously over multiple recording sessions. The public release does not specify the exact number of individual cattle represented in the footage, the precise number of videos contributed by each animal, or the boundaries between individual recording sessions. Stratified three-fold cross-validation in the present analysis was performed at the video level, which ensures that no individual video appears in both training and validation folds but does not guarantee that videos originating from the same animal or recording session are confined to a single fold. We acknowledge in Section 4.4 (Limitations) that grouped (per-animal or per-session) cross-validation would provide a stronger test of biological independence; however, the absence of per-animal identifiers in the public CBVD-5 release precludes such an analysis. Collaborating with the original dataset creators to obtain per-animal identifiers is identified as a methodological priority for any future reanalysis.

**Reduction from the original CBVD-5 collection to the 542 analyzed videos.** The original CBVD-5 collection comprised 687 video clips. Of these, 145 clips were excluded prior to dominant-label consolidation according to the following pre-registered criteria: (i) clips shorter than 1.5 s, providing too few frames for reliable uniform sampling at 16 frames per video; (ii) clips that spanned a behavioral transition (for example, a clip beginning with the animal standing and ending with the animal lying down) such that no single class constituted a clear majority of the frame level annotations; and (iii) clips exhibiting severe motion blur, occlusion by other animals or stall infrastructure, or lighting artifacts that compromised annotation reliability. The remaining 542 clips were retained for analysis.

**Dominant-label consolidation procedure.** Each retained video was assigned a single video-level label equal to the modal class across its frame-level annotations; that is, the class with the highest frame count within the clip. For a clip in which 60 frames were labeled as lying down, 35 frames as rumination, and 5 frames as standing, the dominant label would be lying down, and the rumination content of that clip would be suppressed in the final video-level label. This procedure is the direct cause of the reduction from 13 rumination-containing clips at the frame level (Stage 1 in Table 1) to 6 rumination-dominant clips at the video level (Stages 2–5): 7 clips in which rumination was present but was not the modal frame-level class were assigned to lying down or standing. The same mechanism accounts for the disappearance of foraging (4 clips containing foraging content were all assigned to standing or lying down). We acknowledge that this procedure may have contributed to the observed failure of rumination classification, because rumination that co-occurred with standing or lying was systematically relabeled as the dominant posture. A more biologically faithful multi-label or hierarchical formulation is discussed in Section 2.2 (Proposed Architecture) and Section 4.4 (Limitations) but is outside the scope of the present four-architecture comparison. To make the distinction explicit: Stage 1 in Table 1 reports the original CBVD-5 video-level labels as supplied in the publicly released dataset, whereas Stages 2–5 report the recomputed dominant labels after the consolidation procedure described above. The two are different operations: Stage 1 counts videos originally labeled as each behavior class by the CBVD-5 release, while Stages 2–5 count videos whose *recomputed dominant frame-level label* is each behavior class. The rumination column accordingly differs between the two: 13 videos were originally labeled as rumination by CBVD-5 (Stage 1), of which 7 had rumination as a non-modal frame-level class under our recomputation and were reassigned to standing or lying down in Stages 2–5 (rumination =6). We have verified that the reported Stage 1 counts (326/199/4/0/13, summing to 542) match the publicly released CBVD-5 video-level label distribution, and that the Stages 2–5 counts (330/206/0/0/6, summing to 542) match the output of the dominant-label consolidation procedure implemented in this study; both are internally consistent.

Interpretation of Table 1. Stage 1 reports the original CBVD-5 video-level label distribution, that is, the labels assigned to each video in the publicly released CBVD-5 dataset (column sum = 542 videos; each video carries exactly one original CBVD-5 label, derived from the modal frame-level class in the original release). Stage 1 should not be read as the count of videos whose recomputed dominant label is each behavior class; that count is reported in Stages 2–5 and is obtained by the dominant-label consolidation procedure described in Section 2.1.1. The two operations are deliberately reported side by side so that the distinction between videos originally labeled as a behavior by CBVD-5 (Stage 1) and videos whose recomputed dominant label is that behavior (Stages 2–5) is explicit. For the rumination column in particular, Stage 1 reports 13 videos originally labeled as rumination by CBVD-5, of which 7 had rumination as a non-modal frame-level class under the recomputation applied in this study and were reassigned to standing or lying down in Stages 2–5 (rumination =6); the foraging column similarly reports 4 videos originally labeled as foraging by CBVD-5 that were reassigned to standing or lying down under the present consolidation. The reported counts are consistent with the publicly released CBVD-5 annotations and with the dominant-label consolidation procedure described in Section 2.1.1. The parenthetical value ∼37,863 is the total number of frame-level annotation rows across these 542 videos (34,102 training + 3761 validation in the original CBVD-5 split) and is a different unit from the per-class columns. Stages 2–5 report video-level counts after the dominant-label consolidation procedure described in Section 2.1.1.

#### 2.1.2. Cross-Validation Splits and Class Imbalance

Due to severe class imbalance, the models were trained primarily on the three present classes using 3-fold stratified cross-validation. In each fold, approximately two-thirds of the videos (∼361 videos) were used for training, while the remaining one-third (∼181 videos) served as the validation set. Stratified partitioning kept the relative frequency of each class roughly consistent from one fold to the next, so that no single fold was disproportionately dominated by the majority classes. Minor differences in the number of training videos assigned per fold, i.e., 361, 361, and 362 respectively arose as a natural consequence of 542 not dividing evenly into three equal parts. For the rumination class specifically, the six rumination videos were distributed across the three stratified folds so that each fold contained two rumination videos; consequently, in each fold the model trained on four rumination videos (the two rumination videos from the other two folds) and validated on the two rumination videos held out in that fold.

#### 2.1.3. Preprocessing and Frame Sampling

Each video was processed by uniformly sampling 16 frames across its temporal length. If the total number of frames in a video is *T*, the sampled frame indices are given by Equation (1):

(1) $$i_{k}=\mathrm{round}\;\left(k\cdot \frac{T-1}{N-1}\right),\;k=0,1,\ldots ,N-1,\;\mathrm{with}\;N=16.$$

**Figure 2:**
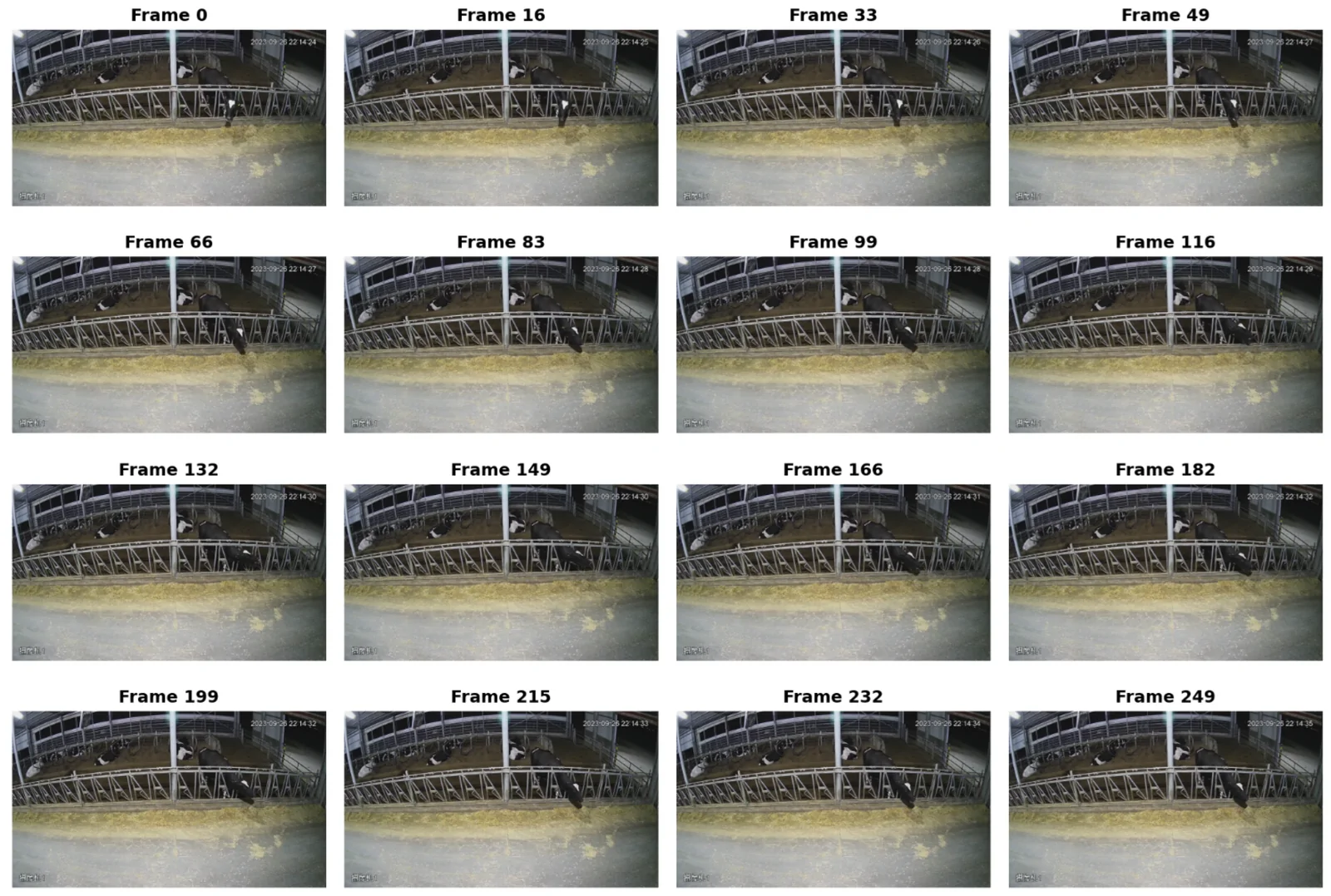
Example of 16 frames uniformly sampled from a video. The example clip shown corresponds to a lying-down instance; the original CBVD-5 label space comprises five behavior classes (standing, lying down, rumination, foraging, drinking water), but after dominant-label consolidation only three classes (standing, lying down, rumination) retained non-zero support in the present analysis, as detailed in Table 1 and Section 2.1.1. The small Chinese-language text in the lower-left corner of each frame is a camera label embedded by the original recording system and is part of the publicly released CBVD-5 footage; it is unrelated to the behavior annotations used in this study.

Equal interval sampling (Figure 2) ensures both the start and end of each behavior are captured. Training frames received stochastic augmentation (random flip, $\pm 10^{\circ }$ rotation, brightness/contrast adjustments) resampled each epoch to prevent memorization. Validation frames received no augmentation. Both subsets were then resized to the backbone’s input resolution and channel-wise standardized using ImageNet statistics (mean [0.485,0.456,0.406], std [0.229,0.224,0.225]), which was the only step applied identically to both sets.

### 2.2. Proposed Architecture

The proposed comparison framework separates spatial feature extraction from temporal aggregation so that the contribution of each component can be evaluated independently. ResNet-50 and EfficientNet-B3 serve as alternative convolutional backbones, while Transformer encoding and temporal averaging provide two distinct strategies for combining frame-level representations. A shared classification head then maps the resulting video representation to the five behavior categories considered in the dataset.

Rationale for the mutually exclusive single-label formulation. The five CBVD-5 behavior categories were treated as mutually exclusive outputs of a single multiclass classifier, following the original annotation scheme of the dataset, in which each frame was labeled with exactly one of the five behavior classes. Biologically, posture (standing, lying down) and activity (rumination, foraging, drinking water) represent different behavioral dimensions, and cattle may ruminate while either standing or lying. The mutually-exclusive single-label formulation was nevertheless retained for three reasons. First, the original CBVD-5 annotations were structured as per-frame exclusive labels, and re-deriving a multi-label or hierarchical ground truth from per-frame data is outside the scope of the present four-architecture comparison. Second, the temporal-aggregation strategies evaluated here (Transformer encoder and temporal averaging) each produce a single feature vector per video, which requires a single prediction target. Third, a multi-label formulation would require additional per-frame or per-segment annotation that the public CBVD-5 release does not provide. We accordingly discuss in Section 4.4 (Limitations) that the single-label formulation may have suppressed rumination events that co-occurred with standing or lying, and we identify a hierarchical formulation in which posture and activity are predicted jointly as a more biologically appropriate target for future work.

#### 2.2.1. CNN Backbone

We evaluated two backbones with different design philosophies that is ResNet-50 and EfficientNet-B3. ResNet-50 uses identity skip connections that bypass intermediate transformations, creating an unobstructed gradient path that stabilizes training even at great depth [7]. For our small training folds (≈360 videos per fold), this stability is essential, whereas without it reliable convergence would be genuinely difficult. EfficientNet-B3 was optimized for a different goal: finding the depth, width, and input resolution that maximize accuracy per parameter, rather than prioritizing gradient flow. A compound scaling coefficient derived from neural architecture search expands all three dimensions simultaneously in a fixed ratio, producing a family of models whose accuracy-efficiency trade-off outperforms networks scaled along any single dimension alone. At its native input resolution of 300×300 pixels, EfficientNet-B3 produces 1536-dimensional frame embeddings that are more information-dense per parameter than the 2048-dimensional embeddings extracted by ResNet-50 at 224×224.

For model evaluation each of the 16 uniformly sampled frames is passed through the selected backbone with its original classification head removed, so that the network functions purely as a feature extractor. The output for each frame is a single embedding vector, e.g., 2048 dimensions for ResNet-50 and 1536 for EfficientNet-B3; stacking these 16 vectors then produces the clip-level matrix $X\in R^{N\times d}$ that is subsequently passed to the temporal aggregation module.

#### 2.2.2. Temporal Modeling Strategies

The sequence *X* is processed by a Transformer encoder with *L* layers, each containing a multi-head self-attention (MHSA) block and a position-wise feed-forward network (FFN). For each head *h* (with $d_{k}=d/H$), Equation (2) projects the input *X* into queries, keys, and values:

(2) $$Q_{h}=XW_{h}^{Q},\;K_{h}=XW_{h}^{K},\;V_{h}=XW_{h}^{V}.$$

The scaled dot-product attention for head *h* is computed using Equation (3):

(3) $$\mathrm{Attn}\left(Q_{h},K_{h},V_{h}\right)=\mathrm{softmax}\;\left(\frac{Q_{h}K_{h}^{⊤}}{\sqrt{d_{k}}}\right)V_{h}.$$

The outputs of all heads are concatenated and linearly projected according to Equation (4):

(4) $$M\left(X\right)=\mathrm{Concat}\left({\mathrm{head}}_{1},\ldots ,{\mathrm{head}}_{H}\right)W^{O}.$$

A residual connection and layer normalization are then applied as shown in Equation (5):

(5) $$X^{\prime }=\mathrm{LayerNorm}\left(X+M\left(X\right)\right).$$

The feed-forward network applies two linear transformations with a GELU activation, as defined in Equation (6):

(6) $$\mathrm{FFN}\left(X^{\prime }\right)=\mathrm{GELU}\left(X^{\prime }W_{1}+b_{1}\right)W_{2}+b_{2}.$$

Another residual connection and layer normalization produce the output of one layer through Equation (7):

(7) $$X^{\prime \prime }=\mathrm{LayerNorm}\left(X^{\prime }+\mathrm{FFN}\left(X^{\prime }\right)\right).$$

After *L* layers, the final encoded sequence is $X_{L}$.

Temporal Averaging. In the temporal-averaging models, the sequence of frame features is averaged across the temporal dimension instead of using a Transformer, according to Equation (8):

(8) $$h_{\mathrm{avg}}=\frac{1}{N}\sum_{t}x_{t}\in R^{d}.$$

This aggregation strategy is simple, computationally efficient, and aims at assessing the added computational value of its counterpart.

#### 2.2.3. Classification Head

For both Transformer and averaging models, the final temporal representation is passed through the linear classification layer in Equation (9):

(9) $$z=W_{c}h_{\mathrm{final}}+b_{c},\;\mathrm{where}\;W_{c}\in R^{C\times d},\;b_{c}\in R^{C},$$

where C=5 denotes the number of behavior classes. The predicted class is $\hat{y}=\arg\max_{c}z_{c}$.

### 2.3. Training Configuration

All four models share a common training protocol fixed before any results were examined. Class imbalance is addressed at the loss level through weighted cross-entropy, with per-fold weights computed as $w_{c}=M/\left(C\cdot f_{c}\right)$, where *M* is the number of training samples in the current fold, C=5, and $f_{c}$ is the observed count of class *c* in that fold. Under this scheme, each training sample contributes to the total loss in inverse proportion to how frequently its class appears; that is, errors on rumination clips carry substantially more weight than errors on standing clips, without removing any videos or synthetically duplicating rare ones.

**Figure 3:**
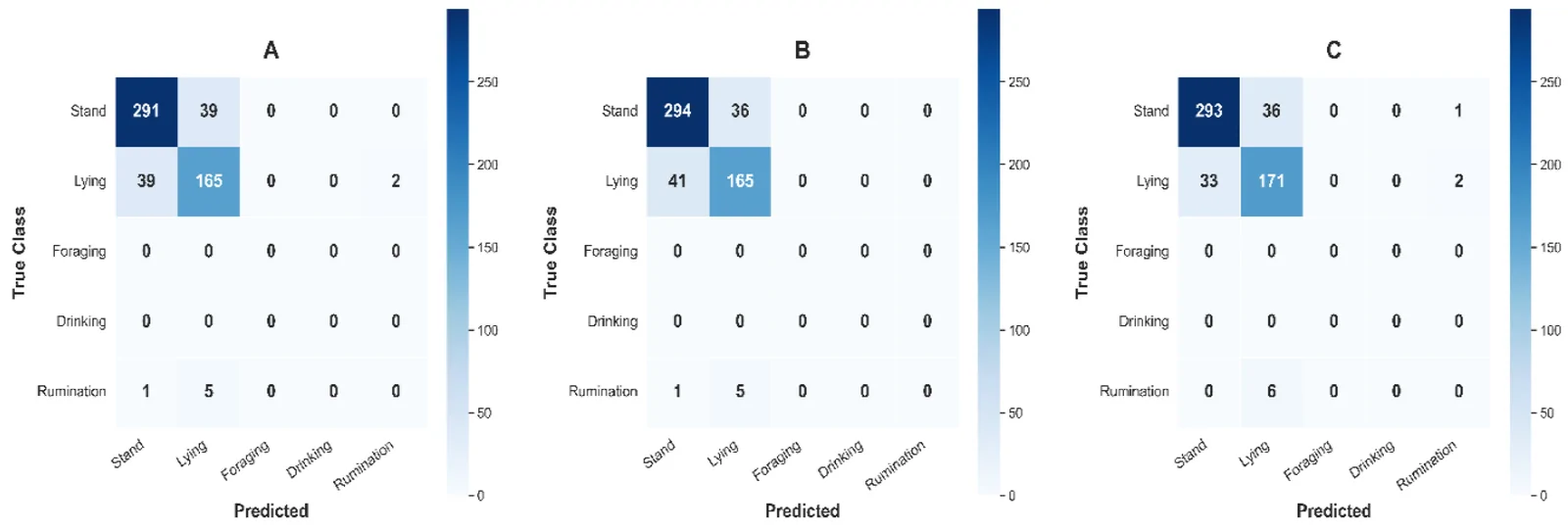
Raw confusion matrices comparing the classification performance of (**A**) Eff-B3 + TF, (**B**) ResNet-50 + TF, and (**C**) Eff-B3 + Avg across different behavioral classes.

Handling of zero-support classes. After dominant-label consolidation, foraging and drinking water have zero support in every training fold, which makes $f_{c}=0$ and the inverse-frequency weight $w_{c}=M/\left(C\cdot 0\right)$ mathematically undefined. In our implementation, zero-support classes were assigned a weight of zero, with the result that they contributed nothing to the loss function during training. The classification head retained all five output logits, and the training loss was implemented as standard five-class weighted softmax cross-entropy without any explicit masking of the logits corresponding to the two absent classes. We accordingly correct the previous statement that “the two zero-support logits received no gradient signal”: under standard softmax cross-entropy, the gradient of the per-sample loss with respect to any logit $z_{c}$ is $\partial L/\partial z_{c}\propto p_{c}\;-$ 1[c=t], where $p_{c}=\exp\left(z_{c}\right)/\sum_{c^{\prime }}\exp\left(z_{c^{\prime }}\right)$ is the softmax probability of class *c* and *t* is the target class; because $p_{c}$ depends on all logits through the normalizing denominator, the absent-class logits can in principle receive non-zero gradients even when their per-sample target weight is set to zero, since no positive target ever appears for them but they still participate in the softmax normalization of the present-class targets. Setting the class weight $w_{t}=0$ for absent classes removes the per-sample contribution of any sample whose target is the absent class, but it does not by itself zero the gradients flowing into the absent-class logits when other classes are present; explicit logit masking would have been required to fully suppress those gradients, and this was not performed in our implementation. In practice, however, since no positive target ever pushed the absent-class logits upward and the loss-minimizing configuration places essentially all probability mass on the present classes, the predicted softmax probabilities for foraging and drinking water remained negligibly small throughout training and at convergence. Predictions of foraging and drinking water therefore remained technically possible by construction (their softmax outputs were non-zero), but were never observed as the arg-max prediction for any of the 542 out-of-fold videos across the four trained architectures, as reflected in the zero off-diagonal entries of the confusion matrices reported in Section 3.2 (Figure 3). The trained classifier therefore effectively behaves as a three-class system over the present classes (standing, lying down, rumination) at the evaluation threshold; the five-class formulation is retained only to preserve the original CBVD-5 label space for compatibility with future dataset extensions in which foraging or drinking water may regain support.

Weighted cross-entropy as one of several imbalance approaches. Weighted cross-entropy with stratified cross-validation is one of several approaches to class imbalance; alternatives include oversampling, undersampling, SMOTE (applicable to tabular but not directly to video data), focal loss, cost-sensitive learning, and anomaly-based detection. Weighted cross-entropy was selected here because it preserves all available data without duplication or synthesis, an important consideration when training samples are scarce, and because it is directly tied to the empirical class frequencies within each fold. We accordingly caution that the failure of rumination classification observed under this specific training strategy should not be generalized to the broader learnability of severely underrepresented behaviors; alternative loss formulations (focal loss), data-level strategies (oversampling), or representation-level strategies (self-supervised pre-training, anomaly detection) may yield different outcomes and are identified in Section 4.4 (Limitations) as methodological alternatives not evaluated here.

Parameter updates were carried out using the Adam optimizer, initialized with a learning rate of $1\times 10^{-4}$ and the standard first- and second-moment decay coefficients of $\beta_{1}=0.9$ and $\beta_{2}=0.999$ respectively. A ReduceLROnPlateau scheduler stepped the learning rate down by half, allowing the optimizer to trade exploration for fine-grained convergence as training progressed. Automatic Mixed Precision (AMP) ran throughout to keep VRAM usage within the available 8 GB and reduce per-epoch wall-clock time. Each fold trained for at most 30 epochs; the checkpoint achieving the highest validation accuracy within that fold was retained for all subsequent evaluation with no weight averaging or other post hoc processing applied. Checkpoint selection on the basis of overall validation accuracy was chosen for two reasons. First, the four architectures were compared under a common protocol, and using a different selection metric for each model would introduce a confound that complicates the cross-architecture comparison. Second, the alternative class-balanced metrics (macro-F1, balanced accuracy) are themselves unstable when one class has only four training videos and two validation videos per fold (the typical rumination split after stratified 3-fold partitioning of the six rumination videos, i.e., 6=4+2 across the three folds; we have corrected the previous wording that referred to “two training samples per fold”, which incorrectly described the validation-side count rather than the training-side count), because a single correct or incorrect rumination prediction swings macro-F1 by a large amount in some folds. Architecture-specific hyperparameters are summarized in Table 2; cross-validation accuracy, pooled classification performance, test-video predictions, calibration, rare-class analysis, and computational efficiency are reported in Table 3, Table 4, Table 5, Table 6, Table 7, and Table 8, respectively. We therefore retained accuracy as the primary selection criterion while reporting class-balanced metrics (balanced accuracy and macro-F1) in the frame-ablation table (Table 9) for completeness. Because accuracy is dominated by the majority classes, the dedicated rare-class analysis in Section 3.7 and the discussion in Section 4.1 report class-ECE and per-class precision/recall separately rather than relying on the aggregate metric. Stratified 3-fold cross-validation was partitioned at video level using random seed 42. Experiments were run on an NVIDIA GeForce RTX 2070 GPU (8 GB VRAM) with 64 GB system RAM, under Python 3.11, PyTorch 2.0.1, and CUDA 11.8.

### 2.4. Experimental Setup and Evaluation Metrics

The experimental protocol was designed to evaluate discrimination, generalization, calibration, and rare-class behavior under the same cross-validation splits. Fold-wise validation and pooled out-of-fold predictions provide complementary estimates of model stability and overall performance. The following subsections define the evaluation strategies, classification metrics, and dedicated rare-class analysis used throughout the Section 3.

#### 2.4.1. Evaluation Strategies

Under severe class imbalance, no single metric gives a complete picture. We therefore evaluated model quality from four distinct perspectives rather than one summary figure.

Throughout training, the highest validation accuracy achieved within each fold determined which model checkpoint was saved. Across the three folds, performance is reported as a mean accuracy with accompanying standard deviation and 95% confidence interval, jointly conveying both the central tendency and the degree of fold-to-fold consistency.

To obtain a generalization estimate covering the full dataset, out-of-fold predictions were pooled across all three folds. Each video contributed exactly one prediction, produced by the best checkpoint from the fold in which that video was held out for validation. No video was therefore ever scored by a model that had trained on it. Collecting these predictions across all 542 videos allowed a complete confusion matrix to be constructed along with per-class precision, recall, and F1-scores.

Ensemble evaluation was considered to exploit diversity among the three-fold models by forming an ensemble for each architecture and averaging the softmax probability outputs from the best checkpoint of every fold. The final prediction is the class with the highest averaged probability.

Confidence, Calibration, and Temporal Stability were evaluated on representative test videos and all OOF predictions. Frame-level metrics included per-frame confidence, frame-to-frame confidence change rate, rolling standard deviation, prediction entropy, and calibration curves. Probabilistic calibration was quantified using expected calibration error (ECE), maximum calibration error (MCE), Brier score, and negative log-likelihood.

#### 2.4.2. Classification Metrics

Macro and weighted averages of precision, recall, and F1-score were computed only over the three present classes (Stand, Lying down, Rumination). The F1-score is defined in Equation (10):

(10) $$F_{1}=2\times \frac{\mathrm{Precision}\times \mathrm{Recall}}{\mathrm{Precision}+\mathrm{Recall}}.$$

#### 2.4.3. Rare-Class Analysis Protocol

To explicitly inspect model behavior under severe class imbalance, a complementary rare-class analysis was performed on the 16-frame OOF predictions of the best-performing model (Eff-B3 + Avg). Following common practice in imbalanced classification, classes whose support in the OOF set did not exceed 106 videos (i.e., support ≤106, corresponding to roughly one fifth of the largest class) were treated as rare classes for the purpose of this analysis. Under this threshold, only rumination (support =6) qualified as a rare class, whereas stand (330) and lying down (206) were treated as majority classes and foraging and drinking water had zero support and therefore were excluded from the per-class breakdown.

For every class we recorded six complementary quantities that together characterize both the discriminative performance and the probabilistic reliability of the model under imbalance: (i) precision, recall, and F1-score computed against the OOF ground truth; (ii) the false-negative count, indicating how many clips of the true class the model missed entirely; (iii) the mean softmax confidence the model produced whenever that class was the true label, which reveals whether the model is over-confident on a class it cannot actually recognize; (iv) the mean probability the model assigned to the true class, which exposes whether the predicted probability mass is being allocated to the correct label rather than merely being high in absolute terms; and (v) class-wise Expected Calibration Error (class-ECE) and Maximum Calibration Error (class-MCE), computed by binning only the predictions whose true label matched the class under inspection. These class-wise calibration metrics complement the global ECE/MCE reported in Section 3.5 by isolating the calibration behavior of each behavioral class individually, which is essential for diagnosing whether miscalibration is concentrated in the rare class or distributed uniformly across all classes.

### 2.5. Frame Ablation Study

To determine the optimal number of temporal samples, a frame ablation experiment was conducted on the three best-performing models (ResNet-50 + TF, Eff-B3 + TF, and Eff-B3 + Avg). For each model, we uniformly sampled 8, 16, and 32 frames per video using the same interpolation formula described in Section 2.1.3. All other settings (input resolution, batch size, training epochs, cross-validation folds) remained identical to the main experiments. For each frame count, OOF predictions were recomputed and the same set of classification and calibration metrics was derived. The frame count that maximized accuracy while minimizing inference time was selected as the default for each model.

### 2.6. Computational Efficiency Benchmarking

Computational costs were measured on the same NVIDIA GeForce RTX 2070 GPU used for training. For each architecture the quantities recorded include the total and trainable parameter counts, the storage size in megabytes of both the model state dictionary and the saved checkpoint from the best-performing fold, a mean per-video inference time in milliseconds, averaged across 50 forward passes on a single video under each frame-count condition, and estimated frames per second, calculated as 16frames×1000ms/s divided by the per-video inference time in milliseconds (that is, the number of input frames the model can process per second when running continuously at 16 frames per video). The values reported in Table 8 are calculated with the formula given above.

All benchmarks used the same environment as training, with gradients disabled and mixed precision off, so that the reported latency figures reflect the computational load a system would actually encounter during real-time deployment rather than the reduced overhead associated with training-mode inference. These measurements let us weigh classification and calibration against the storage and latency constraints of each architecture and frame count.

### 2.7. Use of Generative Artificial Intelligence

Generative artificial intelligence (GenAI) tools, including ChatGPT, QuillBot, and Grammarly (web-based versions), were used during manuscript preparation solely for language polishing, grammar correction, punctuation, and formatting adjustments. These tools were not used to generate original scientific content, data, tables, figures, or graphs, or to assist with study design, data collection, video annotation, model development or training, generation of predictions, calculation of performance metrics, statistical analysis, or interpretation of results. All language edits were reviewed and approved by the authors, who take full responsibility for the content of the manuscript.

## 3. Results and Analysis

This section reports the empirical comparison of the four backbone–aggregation combinations under the common training and evaluation protocol. Results are examined from complementary perspectives, including fold-wise stability, pooled classification performance, calibration, temporal consistency, and computational efficiency. Particular attention is given to the behavior of the models under severe class imbalance and to their complete failure on the rare rumination class.

### 3.1. Cross-Validation Performance

Table 3 reports per-fold peak validation accuracies, means, standard deviations, and 95% confidence intervals. Eff-B3 + Avg had the highest mean (85.60%) but the widest spread (σ=3.45%). ResNet-50 + TF achieved a mean cross-validation accuracy of 84.68% (σ=1.42%), slightly lower than Eff-B3 + Avg (85.60%) but higher than Eff-B3 + TF (84.13%). Notably, ResNet-50 + TF also had the tightest confidence interval, indicating the most stable cross-validation behavior. ResNet-50 + Avg lagged at 75.00%, highlighting the importance of both backbone quality and temporal modeling.

### 3.2. Pooled Classification Performance

Pooled OOF metrics for all four architectures are presented in Table 4. Across overall accuracy, Eff-B3 + Avg led at 85.61%, with ResNet-50 + TF and Eff-B3 + TF following at 84.69% and 84.13% respectively; ResNet-50 + Avg trailed the field by a substantial margin at 75.10%. On the standing class, performance was strong across all models, with Eff-B3 + Avg posting the highest precision at 0.8988 and the strongest F1-score at 0.8933. For the lying-down class, Eff-B3 + TF achieved the best recall at 0.8398 and the highest F1-score at 0.8199, though its precision of 0.8009 was marginally below that of ResNet-50 + TF at 0.8010. Critically, no model successfully classifies any rumination example (F1 = 0 for all), confirming that with only six positive samples in the dataset, even cross-validation cannot enable reliable detection of this rare behavior.

Figure 3 presents the raw confusion matrices for the three strongest models. All three demonstrated strong performance on the two dominant classes, Stand and Lying down, as indicated by the high values along the main diagonal. ResNet-50 + TF correctly classifies the most standing samples (294/330), followed by Eff-B3 + Avg (293) and Eff-B3 + TF (291). For lying down, Eff-B3 + Avg performs best (171/206), ahead of both Transformer models (165 each). Critically, no model correctly classifies any rumination sample (true positive =0 for all models). All six rumination instances are misclassified: predominantly as lying down and occasionally as standing (1 out of 6). The matrices also show negligible confusion with foraging and drinking water, which have zero support in the dataset. The confusion matrices make explicit that the apparently favorable overall accuracy reported in Table 4 is driven almost entirely by correct classification of the two majority postural classes. The absence of any true-positive rumination predictions, visible as a zero in the rumination column of every confusion matrix, is the practical significance takeaway for deployment: any operator consuming the reported accuracy as a deployment-readiness signal would substantially overestimate the model’s coverage of the full CBVD-5 behavior space.

### 3.3. Qualitative Evaluation on a Test Video

As a qualitative demonstration of the models’ ability to generalize to video data, the ensembles formed by averaging the fold-wise best models were tested on a video: Video 10.mp4 (ground truth: lying down). Table 5 presents the full-sequence prediction results for Eff-B3 + TF and ResNet-50 + TF. The per-frame majority vote strategy produced identical predictions to the full-sequence approach. This single-video demonstration serves as an illustrative example of frame-by-frame prediction behavior on a typical lying-down clip. It does not constitute evidence of generalization to independent animals, farms, environments, or behavioral conditions, and should not be interpreted as such. A rigorous generalization evaluation would require an independent test set collected under different recording conditions, which is outside the scope of the present dataset.

All three models correctly classified all 16 frames (16/16 majority vote). ResNet-50 + TF gave the highest confidences (0.9681 sequence, 0.9677 per-frame), followed by Eff-B3 + TF (0.9170, 0.9098) and Eff-B3 + Avg (0.8076, 0.7904). No frame-to-frame label changes occurred, and confidences stayed stable across the clip.

### 3.4. Training Dynamics

**Figure 4:**
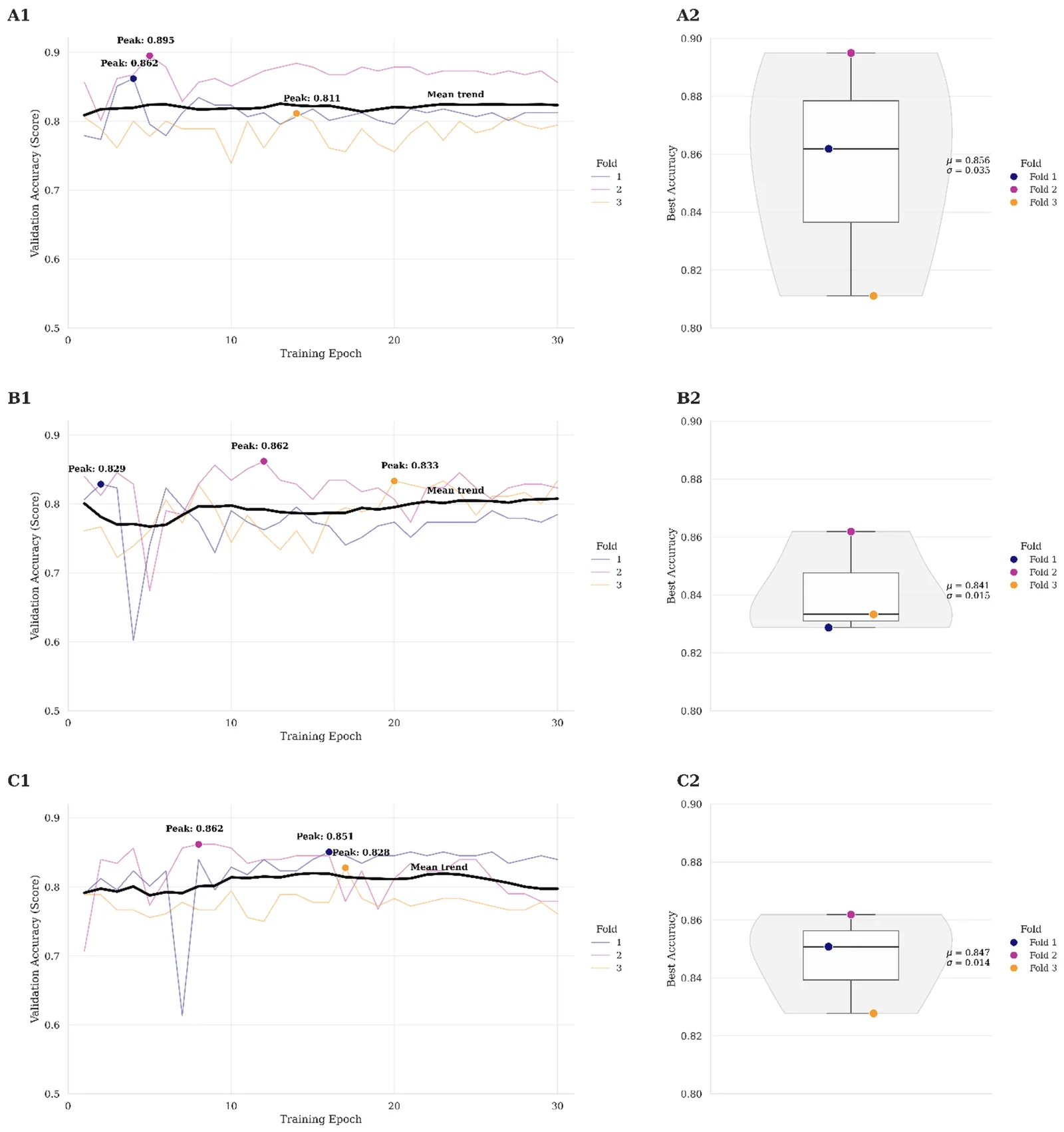
Training dynamics and performance distribution of Eff-B3 + Avg (**A1**,**A2**), Eff-B3 + TF (**B1**,**B2**) and ResNet-50 + TF (**C1**,**C2**).

Figure 4 compares the spatial-temporal feature learning dynamics and cross-validation performance of the top three architectures—Eff-B3 + Avg (A1, A2), Eff-B3 + TF (B1, B2) and ResNet-50 + TF (C1, C2)—evaluated across three folds over 30 training epochs. All models converged stably, but the Transformer variants showed more early oscillation.

Eff-B3 + Avg trained most smoothly, reaching a mean best validation accuracy of 0.856 (peak fold 0.895) with less epoch-to-epoch instability than either Transformer. ResNet-50 + TF achieves comparable performance (μ=0.847) and exhibits the lowest fold-to-fold variability (σ=0.014), indicating robust generalization. Eff-B3 + TF converged reliably but scored lowest (0.841), reflecting that consistent training does not always mean better training.

### 3.5. Confidence and Temporal Stability Analysis

**Figure 5:**
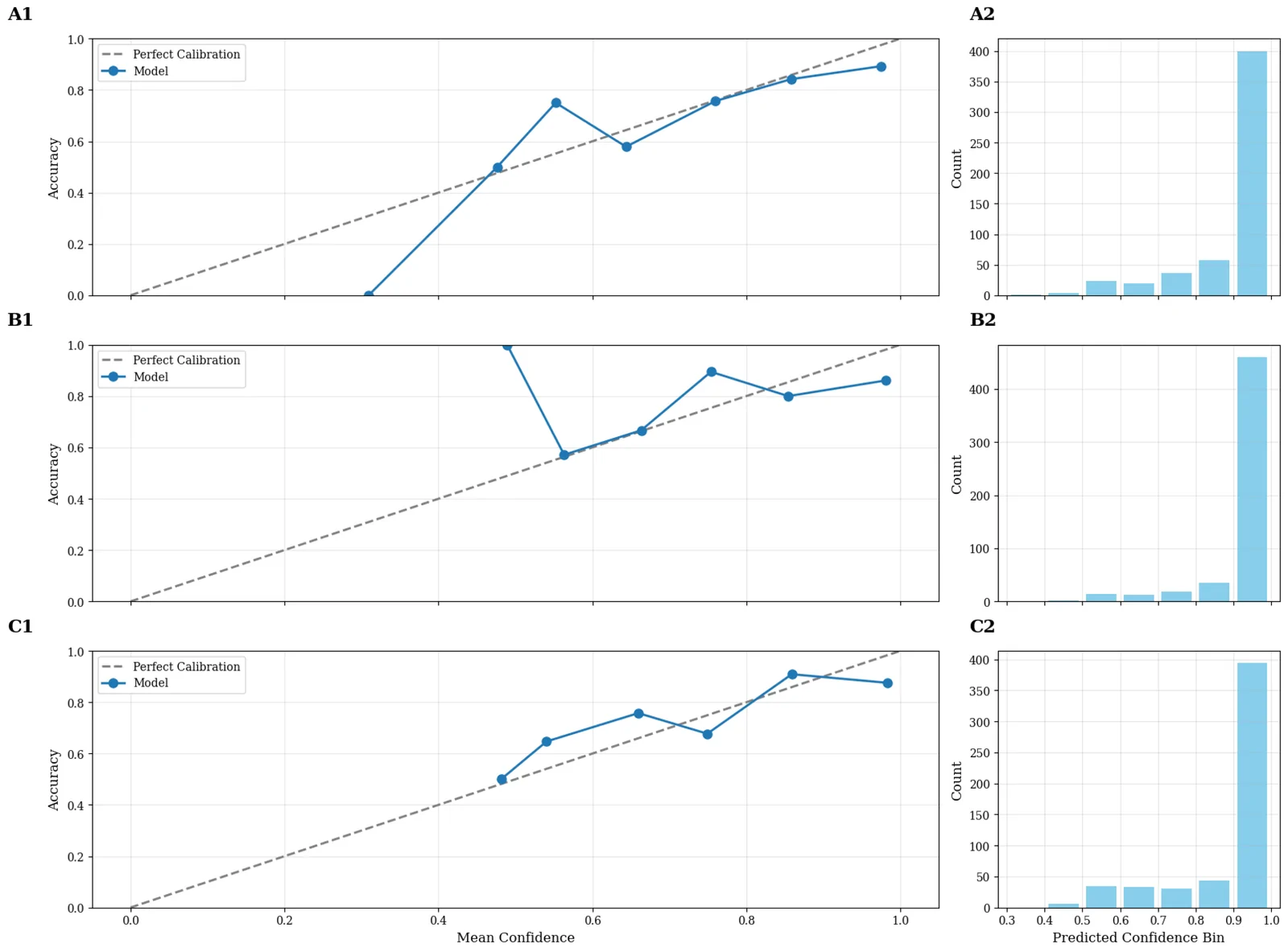
Reliability diagrams (**left**) and confidence histograms (**right**) for Eff-B3 + Avg (**A1**,**A2**), ResNet-50 + TF (**B1**,**B2**), and Eff-B3 + TF (**C1**,**C2**).

Calibration was evaluated on all 542 out-of-fold predictions using maximum softmax confidence and assessed with negative log-likelihood (NLL), multiclass Brier score, expected calibration error (ECE), and maximum calibration error (MCE). The results are summarized in Table 6, with reliability diagrams and confidence histograms shown in Figure 5.

All three models achieved similar accuracy (84.1–85.6%). Eff-B3 + Avg excelled in calibration: lowest NLL (0.6019), lowest Brier (0.2532), smallest ECE (0.0741), and a reliability diagram close to perfect. ResNet-50 + TF had the highest mean confidence (0.945) but the largest MCE (0.511)—a notable high-confidence miscalibration. Eff-B3 + TF was intermediate (ECE 0.0995, MCE 0.1080) but had the worst NLL, indicating less reliable probability alignment.

Confidence histograms confirmed that all three models concentrated the majority of their predictions in the uppermost bin spanning 0.9 to 1.0, with Eff-B3 + TF showing the sharpest clustering in that range. Temporal coherence, assessed on a separate 16-frame test clip, was strong across all models, with no frame-level label change occurring from one timestep to the next, and confidence values held steady throughout the sequence. Considering accuracy, calibration, and temporal consistency together, Eff-B3 + Avg had the most favorable overall profile and is the recommended architecture when probability reliability matters.

### 3.6. Rare-Class Behavior Analysis

Following the protocol defined in Section 2.4.3, the 16-frame OOF predictions of the best-performing model (Eff-B3 + Avg) were dissected at the per-class level to explicitly inspect model behavior under severe class imbalance. The threshold of support ≤106 was applied to the OOF support distribution: stand (330) and lying down (206) exceeded the threshold and were retained as majority classes, while rumination (support =6) was the only class that qualified as rare under this criterion. Foraging and drinking water had zero OOF support and were therefore excluded from the per-class breakdown. The resulting per-class behavior is summarized in Table 7.

The two majority classes behaved as expected: stand achieved F1 =0.8933 with 37 false negatives, and lying down achieved F1 =0.8162 with 35 false negatives, and in both cases the mean probability the model assigned to the true class (0.845 for stand and 0.768 for lying down) was the dominant contributor to the model’s overall confidence, confirming that the high overall accuracy of Eff-B3 + Avg is genuinely supported by accurate probability mass allocation on these classes. Class-ECE for the two majority classes was 0.0660 for standing and 0.0877 for lying down. The standing class-ECE falls below the global ECE of 0.0741, indicating that calibration on the standing class is slightly better than the global average; however, the lying-down class-ECE of 0.0877 is higher than the global ECE of 0.0741, indicating that calibration on the lying-down class is slightly worse than the global average. Both majority-class values remain substantially smaller than the rumination class-ECE of 0.891, confirming that the residual global miscalibration of Eff-B3 + Avg is dominated by the rare class rather than uniformly distributed.

The picture for the rare rumination class is fundamentally different and reveals a failure mode that the global metrics partially obscure. Although the model produced a mean softmax confidence of 0.891 whenever rumination was the true label, only 0.0101 of that probability mass was on average allocated to the rumination class itself. In other words, the model was highly confident on rumination clips, but that confidence was systematically directed at one of the majority classes (predominantly lying down) rather than at the correct label. All six rumination clips were misclassified, giving precision = recall = F1 =0 and a false-negative count of 6. The class-wise calibration metrics quantify this collapse: rumination registered a class-ECE of 0.891 and a class-MCE of 0.972, both an order of magnitude larger than the corresponding values for the majority classes. This confirms that the residual global miscalibration of Eff-B3 + Avg (ECE =0.0741) is not uniformly distributed across classes but is instead almost entirely concentrated in the single rare class. No calibration post-processing such as temperature scaling, which only rescales the global softmax temperature, can repair this failure, because the problem is not a global overconfidence but a structural misallocation of probability mass to the wrong class label.

### 3.7. Computational Efficiency and Frame Ablation

Table 8 compares computational efficiency of the top three architectures under three sampling rates (8, 16, 32 frames) on the same hardware (NVIDIA RTX 2070).

Despite its 73.9 million parameters and 282 MB footprint, ResNet-50 + TF was the fastest model at 17.9 ms per video. This counterintuitive result stems from its lower input resolution of 224×224 pixels: the reduced spatial dimensionality cuts the computational cost of each forward pass enough to offset the overhead of the larger parameter count. Eff-B3 + Avg occupied the opposite corner of the efficiency space, pairing the smallest storage requirement with an inference time of 28.2 ms that still supports real-time processing, while Eff-B3 + TF fell between the two on both storage and speed. For completeness, the Eff-B3 + Avg model contained exactly 10,703,917 trainable parameters, corresponding to an estimated state-dict size of 41.17 MB and a saved checkpoint size of 123.32 MB.

Frame ablation results across 8, 16, and 32 frames per video are consolidated in Table 9. For both EfficientNet-based architectures, 16 frames provided the most favorable observed accuracy-efficiency trade-off. The accuracy difference between 8 and 16 frames for Eff-B3 + Avg was small (0.8542 vs. 0.8561, a difference of 0.19 percentage points), and we do not claim that this difference is statistically or practically significant in the absence of an explicit significance test. Rather, 16 frames is recommended as the configuration that achieved the highest observed accuracy on the EfficientNet-based architectures without the doubled inference cost of 32 frames. The small accuracy differences between frame counts should therefore not be over-interpreted. Where inference latency is the overriding operational constraint and the storage cost of a larger model is acceptable, ResNet-50 + TF operating at 8 frames delivers equivalent classification accuracy at 11.8 ms per video and warrants consideration. Eff-B3 + TF, by contrast, offers no meaningful advantage over either of the above alternatives at any frame count and should not be the default selection for this task. On the basis of these results, 16 frames can be treated as the fixed operating point for both EfficientNet-based architectures, while 8 frames suffice for ResNet-50 + TF without any sacrifice in accuracy.

## 4. Discussion

This section interprets the experimental findings in relation to model complexity, temporal information, calibration, and practical livestock-monitoring requirements. It compares the observed performance with previous studies while accounting for the unusually imbalanced and incomplete class distribution of the present dataset. The discussion also identifies deployment implications, methodological limitations, and directions for future data collection and model development.

### 4.1. Interpretation of Key Findings

Our experiment asked two questions: does EfficientNet-B3 extract better per-frame features than ResNet-50, and does a Transformer improve clip-level classification over averaging? The results cut against the intuition that more complex architectures are inherently better. On a corpus where the two dominant behaviors are visually separable from individual frames, the simplest temporal aggregation strategy (mean pooling) outperformed self-attention on the stronger EfficientNet-B3 backbone (Eff-B3 + Avg at 85.61% vs. Eff-B3 + TF at 84.13%), but fell substantially short of self-attention on the weaker ResNet-50 backbone (ResNet-50 + Avg at 75.00% vs. ResNet-50 + TF at 84.69%, a difference of nearly 10 percentage points in favor of the Transformer). The Transformer therefore recovers a substantial accuracy deficit on the weaker backbone but does not surpass the simpler EfficientNet-based model. Eff-B3 + Avg recorded the highest mean cross-validation accuracy at 85.61% and the best out-of-fold accuracy at 85.61% with 16 frames. Adding a Transformer to the same EfficientNet-B3 backbone dropped accuracy to 84.13% while expanding the parameter count by a factor of four. The ResNet-50 + Transformer combination settled between the two EfficientNet variants at 84.68% cross-validation and 84.69% OOF, while ResNet-50 with temporal averaging fell well short of all three at 75.00%.

The absence of any benefit from Transformer aggregation follows directly from what standing and lying down look like on video. Both behaviors are defined by whole-body posture rather than by motion patterns that unfold across time: a single frame is almost always sufficient for a competent classifier to assign the correct label, and averaging the per-frame embeddings produced by a well-trained backbone yields a clip representation that is robust without being computationally extravagant. The Transformer’s mechanism of selectively relating distant frames to one another only becomes genuinely useful when the diagnostic signal is distributed unevenly across the temporal dimension, which is a condition that simply does not hold for the posture-dominated behaviors that make up the vast majority of this dataset. The practical implication of this efficiency gap is straightforward. The 1.48 percentage point accuracy advantage that Eff-B3 + Avg holds over its Transformer counterpart does not come close to justifying a fourfold parameter expansion from 10.7 M to 42.2 M, nor the 11% inference slowdown from 28.2 ms to 31.4 ms per video. The one scenario in which adding a Transformer does recoup part of its cost is when ResNet-50 being the weaker backbone was paired with attention recovers nearly ten percentage points of accuracy relative to ResNet-50 + Avg, which suggests that self-attention can partially make up for a backbone that produces less informative per-frame features. Even so, the compensated performance still falls short of what the simpler EfficientNet-based model achieves without any temporal attention at all.

Other notable findings include the optimal frame sampling rate: 16 frames provided the most favorable observed accuracy-efficiency trade-off for both EfficientNet-based models. The accuracy difference between 8 and 16 frames was small, and we do not claim that 8 frames were insufficient in an absolute sense; rather, 16 frames achieved the highest observed accuracy without the doubled inference cost of 32 frames. 32 frames added no accuracy gain but nearly doubled inference time. ResNet-50 + TF achieved the same 84.69% accuracy at all three frame counts, indicating its 224×224 input resolution, not temporal sampling, is the limiting factor. For speed-critical use, ResNet-50 + TF at 8 frames runs in just 11.8 ms per video. Calibration favored Eff-B3 + Avg (ECE =0.074). Eff-B3 + TF had higher ECE (0.100) and the worst negative log-likelihood, meaning its probability estimates are less reliable. Most critically, no model detected any rumination. With only six positive rumination videos distributed across the three stratified folds, each fold’s training set contained at most four rumination videos (the two held-out rumination videos from the other two folds), which are too few for the model to learn discriminative features from, even under weighted cross-entropy loss. We have corrected the previous wording that described the per-fold training count as “two”; the correct per-fold training count is four, with the two rumination videos of the held-out fold serving as validation. All rumination clips were misclassified as majority postures. Under such extreme data constraints, none of the four architectures evaluated here was able to learn rumination. Deploying these models for rumination detection in their current configuration would offer no practical value, but this conclusion is specific to the present architectures and training strategies and does not preclude the possibility that alternative approaches (focal loss, anomaly-based detection, or self-supervised pre-training) could learn rumination from a similar sample size.

The dedicated rare-class analysis reported in Section 3.6 sharpens this conclusion further. Treating classes with OOF support ≤106 as rare isolates rumination as the only rare class in the present dataset and reveals a failure pattern that the global metrics alone do not make explicit: the model is not merely inaccurate on rumination (a fact already visible in Table 4) but is confidently wrong, assigning a mean softmax confidence of 0.891 to rumination clips while channeling on average only 1.01% of that probability mass to the true rumination label. The resulting class-ECE of 0.891 is an order of magnitude larger than the global ECE of 0.0741, which confirms that the residual miscalibration of Eff-B3 + Avg is essentially concentrated entirely in the rare class rather than distributed across all classes. From a deployment standpoint this is a more serious failure than low recall alone, because a monitoring system that consumes the model’s confidence scores as a trigger for human review would receive no signal whatsoever that rumination clips are being mishandled. Any practical deployment of these models for behavior monitoring should therefore treat the rumination output as uninformative by default, regardless of the confidence value the model reports, until a substantially larger rumination corpus is available for retraining.

### 4.2. Comparison with Previous Studies

Our 85.61% accuracy is below the 90.33% reported for a TimeSformer baseline on beef cattle [13]. That study reported results over five behavior categories, whereas our corpus contains two classes with zero representation and one class with six examples, a distribution that mirrors the footage a permanently installed barn camera actually captures over a representative sampling period. Evaluating models on such a corpus produces accuracy figures that are inherently lower, but those figures are arguably more predictive of what a deployed system would achieve in practice. We therefore treat our results as a realistic lower-bound benchmark for imbalanced livestock video classification rather than a shortcoming relative to prior work.

### 4.3. Practical Implications for Precision Livestock Farming

From a deployment perspective, the choice among the four architectures reduces to a question of what resource envelope the target hardware imposes. For computational suitability on resource-constrained edge hardware, Eff-B3 + Avg is the recommended architecture: at 41 MB, it fits within the storage budget of low-cost embedded boards; at 28 ms per video, it comfortably supports real-time processing of standard barn camera feeds; and its global ECE of 0.074 indicates that its confidence scores are reasonably calibrated at the aggregate level. We emphasize that computational suitability for edge deployment is a distinct question from demonstrated biological reliability for practical cattle behavior monitoring. The present dataset originates from a single farm and a single fixed camera configuration; two behavioral classes have no training examples, rumination is represented by only six videos, and no independent external validation was performed. We therefore revise the original “unambiguous” deployment recommendation: the Eff-B3 + Avg architecture is computationally suitable for edge deployment on this class of barn camera footage, but biological reliability for general cattle behavior monitoring, including the absent foraging and drinking classes and the underrepresented rumination class, has not been demonstrated and requires additional multi-farm data collection and external validation. For users without EfficientNet-B3 access or with tight memory, ResNet-50 + TF is a fallback for being slightly less accurate, poorly calibrated, and 282 MB in size. With four times more parameters, slower inference, and lower accuracy, Eff-B3 + TF is not recommended for deployment. It represents a combination that offers no operational advantage at any point along the accuracy-efficiency frontier.

The rare-class results add an important caveat to this recommendation. Even when Eff-B3 + Avg is deployed in its recommended configuration (16 frames, 28 ms latency, 41 MB footprint), operators should not interpret the model’s reported confidence on rumination outputs as a reliable signal. The class-ECE of 0.891 on rumination means that confidence values on rumination predictions are essentially uncorrelated with correctness, and any downstream decision rule that thresholds on confidence will fail silently on this class. The original recommendation to route outputs labeled as rumination to manual review has been reconsidered in light of the observation that no rumination video was correctly classified as rumination by Eff-B3 + Avg: such a rule would not have identified any of the false-negative rumination events observed in this study, because the model never assigns the rumination label to any input. A more appropriate failure-detection strategy would be to flag high-confidence predictions on videos whose frame-level visual content includes jaw movement consistent with rumination, detected by a separate anomaly-based or pose-based pre-filter; this is outside the scope of the present architectures but is identified as a methodological requirement for practical deployment. We accordingly withdraw the original manual-review recommendation and replace it with the more cautious statement that, until a substantially larger and more diverse rumination corpus is available for retraining, the deployed model should be treated as a two-class posture classifier (standing vs. lying down), and rumination monitoring should be deferred to dedicated sensing modalities such as accelerometer-based rumination collars rather than video-based classification.

### 4.4. Limitations

The conclusions drawn here carry several boundaries that bear explicit acknowledgment. The dataset is small by machine learning standards, comprising 542 videos from a single farm captured from a fixed camera angle under a limited range of lighting conditions are among the major circumstances that reduce confidence that learned features will transfer to other facilities, breeds, or seasonal contexts. The structural absence of foraging and drinking-water examples means that the models have never been trained to distinguish these behaviors from the two dominant postures, so any deployment that would encounter them in practice requires, at minimum, a dataset that includes them. Most critically, the six-example rumination class makes that label not learnable under the specific weighted-cross-entropy training strategies evaluated in this study; even the widest confidence intervals in the cross-validation results cannot obscure the fact that zero true positives were observed across all four architectures and all three folds. We accordingly revise the previous statement that “zero true positives reflect a data problem rather than a modeling problem”: the present experiments cannot disentangle the contributing factors, and the observed failure is consistent with several potentially interacting causes that are not separable using the present results. Specifically, (i) sample scarcity (only six rumination videos distributed across three folds, giving four training and two validation videos per fold) limits the gradient signal available for the rare class; (ii) the dominant-label consolidation procedure (Section 2.1.1) systematically relabeled rumination that co-occurred with standing or lying as the dominant posture, which may have removed discriminative training signal; (iii) the four evaluated architectures share the same backbone family (ResNet-50 or EfficientNet-B3) and the same general-purpose classification head, and a different architecture (for example, one with explicit temporal jaw-motion modeling) might perform differently; and (iv) the training strategy was restricted to weighted softmax cross-entropy with stratified sampling, and alternative strategies (focal loss, oversampling, anomaly detection, self-supervised pre-training) were not evaluated. We therefore cannot conclude that the rumination failure is exclusively a data problem; it should be interpreted as the joint outcome of sample scarcity, dominant-label consolidation, architecture choice, and training strategy, none of which can be isolated from the present experiments. We emphasize that this finding does not establish a general impossibility of learning extremely underrepresented behaviors under alternative training strategies such as focal loss, oversampling, anomaly detection, or self-supervised pre-training, which were not evaluated here and may yield different outcomes.

The rare-class analysis also makes explicit a methodological limitation that is otherwise easy to overlook: with only six rumination clips, the per-class calibration metrics reported in Table 7 (class-ECE =0.891, class-MCE =0.972) are themselves estimated from a single confidence bin and should be interpreted as indicators of failure rather than as precise calibration estimates. A larger rumination corpus would be needed both to retrain the model and to obtain statistically reliable class-wise calibration estimates. In addition, the threshold of support ≤106 used to designate rare classes is necessarily dataset-dependent; it was chosen here as roughly one fifth of the largest class support and should be revisited as the dataset grows, since a fixed absolute threshold would become progressively more permissive on a balanced corpus.

Methodological alternatives not evaluated in this study but available to subsequent analyses include focal loss as a reweighting alternative to inverse-frequency weighting, oversampling of rare clips as a data-level alternative, anomaly-detection formulations that treat rumination as a deviation from a majority-class baseline, and self-supervised pre-training on unlabeled farm footage followed by supervised fine-tuning on the small labeled set. The present results speak only to the four evaluated architectures under weighted cross-entropy; they do not preclude the possibility that one of these alternative formulations could learn rumination from a comparable sample size. Future cattle-welfare studies could also consider complementary physiological measures, provided they are validated specifically for cattle.

## 5. Conclusions

This study demonstrates that, for posture-dominated cattle behaviors recorded under realistic single-camera barn conditions, a computationally lightweight architecture combining EfficientNet-B3 with temporal averaging provides the most favorable trade-off among classification accuracy, computational efficiency, and probabilistic calibration of the four evaluated configurations. The finding that the simplest temporal aggregation strategy outperformed a more complex Transformer encoder on the stronger backbone, and that the Transformer only partially recovers the accuracy deficit on the weaker ResNet-50 backbone, challenges the assumption that architectural complexity is inherently beneficial for livestock video classification and supports the deployment of efficient models on resource-constrained edge devices.

The complete failure to recognize rumination, despite explicit weighted-loss and stratified-sampling countermeasures, establishes a practical lower bound on what can be achieved with six positive examples and indicates that aggregate-accuracy metrics can mask severe per-class failures. The dedicated rare-class analysis revealed that this failure is not merely a low-recall problem but a confidently wrong problem, with the model assigning a mean softmax confidence of 0.891 to rumination clips while channeling only 1.01% of probability mass to the true rumination label. These results are directly applicable to two-class posture-monitoring deployments on edge hardware; they are not applicable to rumination detection, multi-activity recognition, or cross-farm generalization without substantial additional data collection.

The methodological contribution of this work lies in providing a deployment-oriented evaluation framework that combines discrimination, calibration, temporal consistency, frame-count sensitivity, and rare-class analysis within a single cross-validated protocol, an approach that can be transferred to other livestock video classification problems in which rare behaviors are of practical concern. Subsequent work should prioritize multi-farm data collection with per-animal identifiers, evaluation of alternative imbalance-handling strategies (focal loss, oversampling, anomaly detection, self-supervised pre-training), and a hierarchical formulation that decouples posture from activity, in order to test whether the rumination failure observed here is intrinsic to the dataset size or to the training strategy.

## Figures and Tables

**Table 1:** Distribution of video-level labels in the original dataset and videos evaluated in out-of-fold (OOF) predictions.

Stage	Description	Stand	Lying Down	Foraging	Drinking	Rumination	Total
1	Original CBVD-5 video-level labels (modal frame-level class per the publicly released CBVD-5 dataset; ∼37,863 frame-level annotation rows)	326	199	4	0	13	542
2	Video-level dominant label after preprocessing	330	206	0	0	6	542
3	Videos in 3-fold splitting	330	206	0	0	6	542
4	Combined OOF $y_{\mathrm{true}}$ count	330	206	0	0	6	542
5	Support in classification report	330	206	0	0	6	542

**Table 2:** Hyperparameter summary for each architecture.

Model	Backbone	Input Res.	Feature Dim.	Temporal Modeling	Batch Size
ResNet-50 + TF	ResNet-50	224×224	2048	Transformer (2L, 8H)	4
Eff-B3 + TF	EfficientNet-B3	300×300	1536	Transformer (2L, 8H)	2
Eff-B3 + Avg	EfficientNet-B3	300×300	1536	Temporal averaging	2
ResNet-50 + Avg	ResNet-50	224×224	2048	Temporal averaging	2

**Table 3:** Cross-validation accuracy comparison among the four models.

Model	Fold 1 Acc.	Fold 2 Acc.	Fold 3 Acc.	Mean ± Std	95% CI
ResNet-50 + TF	85.08%	86.19%	82.78%	84.68% ± 1.42%	[83.09%, 86.29%]
Eff-B3 + TF	82.87%	86.19%	83.33%	84.13% ± 1.47%	[82.47%, 85.79%]
Eff-B3 + Avg	86.19%	89.50%	81.11%	85.60% ± 3.45%	[81.70%, 89.51%]
ResNet-50 + Avg	69.06%	76.24%	80.00%	75.00% ± 5.00%	[69.97%, 80.24%]

**Table 4:** Pooled per-class classification metrics using OOF predictions. Macro averages are calculated only over the three present classes (Stand, Lying down, Rumination).

Model	Class	Precision	Recall	F1-Score	Support
ResNet-50 + TF	Stand	0.8750	0.8909	0.8829	330
	Lying down	0.8010	0.8010	0.8010	206
	Rumination	0.0000	0.0000	0.0000	6
	Accuracy	—	—	0.8469	542
Eff-B3 + TF	Stand	0.8957	0.8848	0.8902	330
	Lying down	0.8009	0.8398	0.8199	206
	Rumination	0.0000	0.0000	0.0000	6
	Accuracy	—	—	0.8413	542
Eff-B3 + Avg	Stand	0.8988	0.8879	0.8933	330
	Lying down	0.8028	0.8301	0.8162	206
	Rumination	0.0000	0.0000	0.0000	6
	Accuracy	—	—	0.8561	542
ResNet-50 + Avg	Stand	0.7594	0.7594	0.7594	330
	Lying down	0.7594	0.7594	0.7594	206
	Rumination	0.0000	0.0000	0.0000	6
	Accuracy	—	—	0.7510	542

**Table 5:** Ensemble inference results on a single test video (Video 10.mp4, GT: lying down) using full 16-frame prediction.

Model	Video ID (GT)	Full-Seq Prediction (Confidence)	Majority Vote (Correct/Total)	Avg Frame Confidence
Eff-B3 + Avg	Video 10 (lying down)	Lying down (0.8076)	16/16	0.7904
ResNet-50 + TF	Video 10 (lying down)	Lying down (0.9681)	16/16	0.9677
Eff-B3 + TF	Video 10 (lying down)	Lying down (0.9170)	16/16	0.9098

**Table 6:** Calibration and confidence metrics for the three best-performing models on OOF predictions (542 samples).

Model	OOF *n*	Accuracy	Mean Conf.	Conf.–Acc. Gap	NLL	Brier Score	ECE (10 bins)	MCE (10 bins)
Eff-B3 + Avg	542	0.8561	0.9124	0.0563	0.6019	0.2532	0.0741	0.3093
ResNet-50 + TF	542	0.8469	0.9451	0.0983	0.6968	0.2817	0.1125	0.5107
Eff-B3 + TF	542	0.8413	0.9070	0.0657	0.7162	0.2785	0.0995	0.1080

**Table 7:** Per-class rare-behavior analysis on 16-frame OOF predictions of Eff-B3 + Avg. Rare classes (support ≤106) are marked; foraging and drinking water have zero support and are omitted from the per-class breakdown. “Mean conf.” is the mean softmax confidence the model produced when the row class was the true label; “Mean P(true)” is the mean probability the model assigned to the true class.

Class	Support	Precision	Recall	F1	FN Count	Mean conf.	Mean P(true)	Class-ECE	Class-MCE	Rare?
Stand	330	0.8988	0.8879	0.8933	37	0.9296	0.8452	0.0660	0.5356	No
Lying down	206	0.8028	0.8301	0.8162	35	0.8855	0.7680	0.0877	0.2376	No
Foraging	0	—	—	—	—	—	—	0.0029	0.2297	No (zero support)
Drinking water	0	—	—	—	—	—	—	0.0028	0.2083	No (zero support)
Rumination	6	0.0000	0.0000	0.0000	6	0.8914	0.0101	0.8914	0.9718	Yes

**Table 8:** Computational efficiency comparison (16 frames per video).

Model	Total Params	Model Size (MB)	Checkpoint Size (MB)	Inference Time (ms/video)	Est. FPS
Eff-B3 + Avg	10.7 M	41.2	123.3	28.2	567
ResNet-50 + TF	73.9 M	282.1	846.1	17.9	896
Eff-B3 + TF	42.2 M	161.3	483.7	31.4	510

**Table 9:** Frame ablation results on 542 OOF samples.

Model	Frames	Accuracy	Balanced Acc.	Macro F1	Weighted F1	NLL	Brier	ECE	MCE	Overconf. Gap
Eff-B3 + Avg	8	0.8542	0.5704	0.5683	0.8521	0.6057	0.2551	0.0803	0.3071	0.0585
	16	0.8561	0.5727	0.5698	0.8541	0.6019	0.2532	0.0741	0.3093	0.0563
	32	0.8524	0.5694	0.5670	0.8503	0.6028	0.2541	0.0773	0.3091	0.0600
ResNet-50 + TF	8	0.8469	0.5640	0.5613	0.8420	0.7006	0.2832	0.1118	0.5635	0.0983
	16	0.8469	0.5640	0.5613	0.8420	0.6968	0.2817	0.1125	0.5107	0.0983
	32	0.8469	0.5640	0.5613	0.8420	0.6958	0.2812	0.1149	0.5116	0.0983
Eff-B3 + TF	8	0.8339	0.5563	0.5535	0.8311	0.7183	0.2813	0.0929	0.1079	0.0728
	16	0.8413	0.5609	0.5586	0.8383	0.7162	0.2785	0.0995	0.1080	0.0657
	32	0.8376	0.5583	0.5559	0.8346	0.7195	0.2797	0.1024	0.1422	0.0688

## Data Availability

The CBVD-5 dataset used in this study is publicly available on Kaggle at https://www.kaggle.com/datasets/fandaoerji/cbvd-5cow-behavior-video-dataset (accessed on 2 September 2026). Code and trained model checkpoints can be made available upon reasonable request to the corresponding author.
